# 2268 Utilizing digital pedagogy to build communication skills in predoctoral training programs

**DOI:** 10.1017/cts.2018.234

**Published:** 2018-11-21

**Authors:** Karen M. Weavers, Becca Gas

**Affiliations:** Mayo Clinic

## Abstract

OBJECTIVES/SPECIFIC AIMS: A key factor for success in science is the ability to communicate clearly and succinctly using language appropriate to the audience. Most predoctoral training programs offer opportunities for students to build oral and written communication skills at local and national conferences. However, this rarely provides specific feedback and tends to be episodic. The Mayo Clinic Center for Clinical and Translational Science (CCaTS) has developed an environment for deliberate practice of presentation skills within a weekly Works in Progress and Journal Club session using a learning management system, Blackboard Collaborate. The learning management system captures the presentation that can then be viewed by the student. Watching yourself give a presentation is a powerful learning tool. The learning objectives of the sessions provide students deliberate practice to: (1) Build critical presentation skills for a 1-minute elevator talk, a 2-minute poster overview, a 10-minute oral presentation of your science to a science audience and to a non-science audience. (2) Develop constructive reviewer skills by completing peer reviews of presentations. (3) Develop critical thinking skills to ask thought provoking questions during presentations. By utilizing a curriculum that offers video-recording for reflection and self-evaluation, Mayo Clinic CCaTS has developed an environment in which predoctoral students are encouraged and supported to constantly hone their presentation skills. METHODS/STUDY POPULATION: All CCaTS predoctoral students are asked to prepare presentations in several formats for the weekly 1-hour session. The students’ presentations of their science or journal articles are recorded and saved within Blackboard; a link is provided for the student to review personally, with a mentor, and with the Education Coordinator to discuss the strengths and weaknesses of the presentation. During each session, faculty facilitators encourage students to ask thought provoking questions, and student reviewers are assigned to provide critical and constructive written feedback to the presenter. Sessions providing tools and guidelines for constructive feedback and developing critical and constructive questions are regularly interspersed. RESULTS/ANTICIPATED RESULTS: By reviewing a video recording of their presentations, CCaTS predoctoral students get the opportunity to self-evaluate their performance as an audience member. By going through this process of preparing, presenting, reflecting on their presentations, and discussing their strengths and weaknesses with mentors and classmates, the students gain both powerful presentation skills and methods to improve their delivery and reviewer skills. DISCUSSION/SIGNIFICANCE OF IMPACT: Successful scientists, whether in academia or industry, have the ability to communicate their science clearly using appropriate and common language specific to each audience they present to. By utilizing a curriculum that offers video-recording for reflection and self-evaluation, Mayo Clinic CCaTS has developed an environment in which predoctoral students are encouraged and supported to constantly hone their presentation skills.

